# Toward Best Practices for Controlling Mammalian Cell Culture Environments

**DOI:** 10.3389/fcell.2022.788808

**Published:** 2022-02-21

**Authors:** Shannon G. Klein, Alexandra Steckbauer, Samhan M. Alsolami, Silvia Arossa, Anieka J. Parry, Mo Li, Carlos M. Duarte

**Affiliations:** ^1^ Red Sea Research Center (RSRC) and Computational Bioscience Research Center (CBRC), King Abdullah University of Science and Technology, Thuwal, Saudi Arabia; ^2^ Biological and Environmental Science and Engineering Division (BESE), King Abdullah University of Science and Technology, Thuwal, Saudi Arabia

**Keywords:** batch culture, reproducibility, pH, oxygen, carbon dioxide, stem cells, cancer cells

## Abstract

The characterization, control, and reporting of environmental conditions in mammalian cell cultures is fundamental to ensure physiological relevance and reproducibility in basic and preclinical biomedical research. The potential issue of environment instability in routine cell cultures in affecting biomedical experiments was identified many decades ago. Despite existing evidence showing variable environmental conditions can affect a suite of cellular responses and key experimental readouts, the underreporting of critical parameters affecting cell culture environments in published experiments remains a serious problem. Here, we outline the main sources of potential problems, improved guidelines for reporting, and deliver recommendations to facilitate improved culture-system based research. Addressing the lack of attention paid to culture environments is critical to improve the reproducibility and translation of preclinical research, but constitutes only an initial step towards enhancing the relevance of *in vitro* cell cultures towards *in vivo* physiology.

## Introduction

Mammalian cell cultures have been a foundational resource in almost every biomedical research program since the 1990s (Petricciani, 1995; Hu and Aunins, 1997; Merten, 2006). The use of mammalian cell cultures as preclinical models ranges from the characterization of *in vivo* physiological mechanisms and manipulation of disease-related pathways to the maintenance of stem cells for therapeutic purposes. Culture systems are used to maintain cells in a state that mimics *in vivo* physiological conditions (Papkovsky, 2004; Michl et al., 2019), ensuring the clinical compatibility of experimental findings. Physiological conditions in mammalian cell cultures typically aim to mimic conditions in extracellular fluids, including temperature, typically adjusted to 37°C, O_2_ to 18.6%–20.9%, CO_2_ to 5%, and pH adjusted to 7.4 units (Wenger et al., 2015). Maintaining relevant physiological conditions in cell cultures is of paramount importance to ensure the reproducibility of published findings and the translational relevance of experimental data to clinical applications. Yet, inadequate reproducibility of experimental findings in biomedical research is an increasingly well-recognized problem (Begley, 2013; Collins and Tabak, 2014), contributing to delays in drug discovery and therapies (Freedman et al., 2015).

Best-practice guidelines are used to ensure standards in biomedical research, encompassing multiple aspects of the research practice (Baust et al., 2017), but have not yet included comprehensive standards for the reporting or control of environmental conditions in cell-culture systems. The most common approach to *in vitro* cell culture is when cells are grown either in suspension or as adherent monolayers in standard media within tissue culture flasks (defined here as “standard batch culture”). This approach is most popular since it reliably induces the proliferation of cells, is affordable, and scalable in terms of the possible number of biological replicates and treatments. Although the standard batch culture of cells meets the critical need for continuous sources of biological material, most biomedical researchers using standard culture systems acknowledge that they are limited in their capacity to maintain cell homeostasis within the physiological limits experienced *in vivo* (Place et al., 2017; Al-Ani et al., 2018; Hirsch and Schildknecht, 2019). Despite this awareness and the scope for substantial environmental variability during the standard batch culture of cells (Michl et al., 2019), recent assessments show that the majority of research papers rely on nominal set points and fail to directly verify and report environmental parameters (Al-Ani et al., 2018; Michl et al., 2019; Klein et al., 2021b). Standard batch culture systems undergo substantial environmental changes owing to cell metabolic activity (Balin et al., 1976; Naciri et al., 2008; Vallejos et al., 2010; Pradhan et al., 2012; Al-Ani et al., 2018; Michl et al., 2019), with pH declines reaching 0.9 units, O_2_ levels declining down to 0.95%, and CO_2_ values reaching up to 10.45% (Klein et al., 2021b). In light of these reported changes, the apparent reliance on nominal set points to ensure physiological relevance and reproducibility in biomedical research requires urgent reconsideration. Although Eagle (1971) first highlighted environmental drift in standard batch cultures almost 50 years ago, we are aware of only three papers that collectively measured and reported O_2_ or CO_2_ regimes in *in vitro* cultures of mammalian cells (Balin et al., 1976; Naciri et al., 2008; Vallejos et al., 2010). Inadequate control and reporting of environmental conditions in cell cultures is, therefore, a candidate contributor to irreproducibility in basic and preclinical biomedical research.

Here, we provide guidelines for the reporting and control of environmental conditions in cell culture systems, with a focus on metabolic gases (O_2_ and CO_2_) and the associated acid-base balance driving pH. We (i) raise awareness of the imperative to control and monitor cell culture environments in biomedical research, (ii) propose short- and long-term standards for control, monitoring, and reporting with consideration of resource availability, and (iii) highlight the steps needed for these recommendations to be achieved. We outline the most common problems resulting in uncontrolled environmental conditions and associated confounding factors, and then provide a range of solutions. We also supply a reporting workflow that ensures improved standards for the reporting and control of culture environments to enhance reproducibility and progress in biomedical research.

## The Problems

### Environmental Instability in Cell Culture Media

Documented reports of deoxygenation and disruptions to acid-base stability in culture media caused by cellular metabolism equate to a median pH shift of 0.425 units and a median O_2_ shift of 10.6% from target (nominal) values (see, Klein et al., 2021b). Cells are capable of buffering against extracellular reductions in pH to maintain alkaline pH of the cytoplasm (Johnson et al., 1976; L’allemain et al., 1984; Lindström and Sehlin, 1984; Pouyssegur et al., 1985; McBrian et al., 2013). However, such mechanisms (e.g., Na^+^/H^+^ antiporters or histone deacetylation) consume cellular energy and can alter gene transcription and reduce cellular growth through changes in the acetylation state of chromatin (Boron and Russell, 1983; Bowen and Levinson, 1984; Boron, 2004). Changes in dissolved gases are also well known to substantially affect cellular physiology. Besides the role of O_2_ in affecting the most fundamental characteristics of *in vitro* cell cultures (Packer and Fuehr, 1977), including the dependence of cellular metabolism on O_2_ (Ast and Mootha, 2019), deoxygenation can also activate the hypoxia-inducible factor (HIF) transcription system, which triggers the expression of most genes responsible for cellular adaptation to varying O_2_ levels (Semenza et al., 1991; Wang et al., 1995; Semenza, 2012). Minor deviations in dCO_2_ can also induce a wide range of cellular responses (Bumke et al., 2003; Kikuchi et al., 2017; Kikuchi et al., 2019), affecting the function of biomolecules and the proteome (Duarte et al., 2020).

The impacts of compromised acid-base stability and O_2_ delivery on cellular responses during *in vitro* cell culture are not confined to theory (see syntheses by; Ast and Mootha, 2019; Keeley and Mann, 2019; Klein et al., 2021b). Briefly, for instance, Michl and others (2019) showed that cellular growth of three colorectal cell-lines (NCI-H747, DLD1, Caco2) was optimal at pH 7.4, but when medium pH deviated from 7.4 by > 0.3 units all three cell lines exhibited reduced rates of proliferation. Medium acidification during *in vitro* cell culture can also initiate pro-inflammatory signaling responses in human aortic smooth muscle cells (Tomura et al., 2005) and cells of the human nucleus pulposus (Gilbert et al., 2016). A transcriptomic study, focusing on human fibroblasts, revealed that reductions in medium pH (to pH 6.7) modulated the expression of 2,068 genes (out of 12,565) by more than two-fold after only 24 h of culture (Bumke et al., 2003). Constraining O_2_ availability during *in vitro* cell culture appears to be similarly crucial. For example, HepG2 cultures at confluence rapidly depleted O_2_ levels to <1% to self-inflict a switch from oxidative phosphorylation to glycolysis, despite being cultured in incubator conditions providing ambient atmospheric O_2_ levels (18.6%–20.9%) (Wolff et al., 1993). These findings are highly consistent with observations of other cell types, including human hepatocytes (Ng et al., 2014) and rat renal mesangial cells (Metzen et al., 1995), where perturbed O_2_ levels correlated with anomalous cellular responses (Keeley and Mann, 2019). Although limited, some evidence suggests that variable culture environments can also affect the reproducibility of cell culture experiments. Indeed, barcoding experiments showed that cancer cell-line evolution occurred from positive clonal selection that was highly sensitive to culture conditions (Ben-David et al., 2018). Further experiments testing the cell-line strains against anti-cancer compounds uncovered disparate drug responses, although the exact sources of instabilities in culture environments that promoted cell-line heterogeneity were not resolved (Ben-David et al., 2018).

### Factors Contributing to Environmental Instability in Cell Culture Media

Most commercial media contain buffering systems that act only to regulate pH, whereas levels of dissolved O_2_ and CO_2_ are regulated by atmosphere re-equilibration. The initial stability of medium pH is typically achieved by mimicking the physiologically relevant CO_2_/HCO_3_^−^ buffering system (Michl et al., 2019). Most media formulations contain a known concentration of HCO_3_^−^, which upon exposure to an incubator that nominally maintains a CO_2_-rich atmosphere (typically 5% CO_2_ in air), equilibrates to spontaneously produce H^+^ ions and stabilize pH (Supplementary Box S1). Although the CO_2_/HCO_3_^−^ buffer system is indeed the primary physiological buffering system in mammalian fluids (Boron, 2004; Michl et al., 2019), standard cell cultures lack the regulatory systems (e.g., changes in respiratory rate, vascular remodeling, renal control of HCO_3_^−^ and H^+^) present in the mammalian body. Such systems regulate for the changes in dissolved gases, waste products (e.g., lactic acid), and H^+^ ions involved in cellular metabolism, thereby achieving conditions that maintain homeostasis (Supplementary Box S1). Although consistent observations of pH instability in standard cell cultures prompted the use of additional exogenous buffers in media formulations to enhance medium buffering capacity (Eagle, 1971), such approaches can, in some cases, promote unpredictable changes in pH and introduce confounding artifacts (Michl et al., 2019, see below).

In the case of O_2_, medium deoxygenation is caused by the disparity between rates of O_2_ consumption via cellular metabolism and the replenishment of O_2_ at the air-medium interface (Place et al., 2017; Al-Ani et al., 2018). Specifically, O_2_ first dissolves at the air-medium interface and then diffuses through the liquid (at least several millimeters) to reach and oxygenate cell microenvironments (Place et al., 2017) even as cells undergo exponential growth. This is contrary to *in vivo* physiology, where most cells exist within 100 µm to the nearest capillaries that replenish O_2_ and act to remove excess CO_2_. Besides the role of CO_2_ in affecting medium acid-base chemistry, levels of CO_2_/HCO_3_^−^ readily diffuse across cell membranes to moderate intracellular pH (Gutknecht et al., 1977), act as metabolic inhibitors, and may induce complex transcriptional responses (Cottier et al., 2012; Follonier et al., 2013), and signal other critical reactions (see, Blombach and Takors, 2015). In concert, these processes interact to create a changing environmental gradient from the surface of the medium down to the microenvironment of the cells (Place et al., 2017). The effect of unstirred medium layers also presumably determines the delivery of nutrients/growth factors and the removal of other metabolic waste products (e.g., lactic acid), which can also act to directly and indirectly moderate environmental variation (Michl et al., 2019).

Changes in the culture environment may also initiate complex feedback mechanisms, where cellular responses to variations in the culture environment could, in turn, inflict greater intrusions of environmental stability and promote unpredictable outcomes. For instance, perturbations to dissolved O_2_ levels in culture medium can induce cells to switch away from oxidative phosphorylation towards anaerobic glycolysis (Wolff et al., 1993), leading to large accumulations of lactic acid that force medium acidification (Michl et al., 2019). Another example lies in the role of carbonic anhydrases (CA), which catalyze the hydration of CO_2_. Švastová and others showed that medium deoxygenation in cell cultures of human cancer cell lines induced the expression and activity of carbonic anhydrases, which resulted in enhanced acidification of the culture medium (Švastová et al., 2004).

### Lack of Detailed Methodological Reporting

The lack of monitoring and reporting of environmental conditions in cell culture-systems is a pervasive, but under-recognized problem (Hunter, 2017; Al-Ani et al., 2018; Michl et al., 2019; Klein et al., 2021a; Klein et al., 2021b). A recent synthesis examining this problem sub-sampled 688 papers published between 2014 and 2019 and found that most papers reported the medium manufacturer, but only one third reported the type of culture system utilized and 42% reported temperature and CO_2_ incubator settings (Klein et al., 2021b). Another post-publication analysis reported that less than half of studies published in *Cancer Research* and *Nature* in the third quarter of 2017 described the brand of medium, and only one-tenth declared the medium-buffering regime (Michl et al., 2019). Even when protocols are declared, there is an unfortunate prevalence of papers stating, “as previously described by ref. (x),” which often leads to a chain of citations that generate confusion as to the specific procedures, reagents, and materials involved (Freedman et al., 2015). In cases where environmental parameters are measured, these are often not reported. The apparent under appreciation of reporting measured environmental parameters is exemplified by published bioreactor experiments that report only the target levels of environmental parameters (e.g., Karst et al., 2016; Abecasis et al., 2017). Indeed, these systems, by design, typically require consistent monitoring of the controlled parameters via a feedback loop to achieve the desired control.

### Failure to Monitor Mammalian Cell Culture Environments

A recent synthesis revealed that despite differences in cell type, medium formulation, and buffering components, all investigated standard batch cultures exhibited environmental drift after only a few days of culture (Klein et al., 2021b). Despite this, less than 0.05% of studies monitored pH, CO_2_, or O_2_ levels in cell cultures. Klein et al. (2021a) reported median declines in dissolved O_2_ down to 7.3%, and increases in dissolved CO_2_ to values ranging from 7.5% to 9.5%, compared to the nominal O_2_ and CO_2_ targets of 21% and 5%, respectively. The reported median decline in pH was 0.43 units, but in some particularly extreme cases, cell metabolic activity promoted pH reductions that approached one pH unit (Eagle, 1971) and dissolved O_2_ decreased down to 0.95% (Vallejos et al., 2010). In such extreme cases, variations in culture conditions may resemble levels consistent with hypercapnia and hypoxia rather than conditions typical of *in vivo* extracellular fluids, although *in vivo* environments vary considerably among selected tissues (Ast and Mootha, 2019). It is implicitly assumed that culture temperature is controlled at 37°C and thus, incubator temperatures were only reported in 42% of papers between 2014 and 2019 (Klein et al., 2021b). However, a number of studies used different culture temperatures (Brinkhof et al., 2015; Xu et al., 2017), which highlights the need to declare and monitor the incubation temperature.

### Artefacts Introduced by Forcing of Non-Physiological Controls

Numerous approaches are used to control culture conditions, either to maintain physiological conditions or to test the effects of departures from those conditions (e.g., hypoxia; Grayson et al., 2006; acidosis; Kikuchi et al., 2017). However, some approaches misrepresent *in vivo* physiology and in doing so, inadvertently introduce artefacts and biases that could compromise reproducibility and relevance of the study to cellular function in the living organism. For instance, NVBs (e.g., HEPES, PIPES, or MES) are used to enhance acid-base stability of medium because the physiological HCO_3_^−^/CO_2_ buffering system can exhibit high volatility and a weak buffering capacity (Supplementary Box S1). However, it is vital to consider how NVBs introduce active molecules and acid-base reactions absent from mammalian fluids and existing evidence, although limited, indicates that NVBs could induce toxicity and anomalous cellular responses in particular cell types (Good et al., 1966; Lepe-Zuniga et al., 1987; Hanrahan and Tabcharani, 1990; Stea and Nurse, 1991). In particular, HEPES is commonly included in commercial media formulations, yet emerging reports demonstrate a range of possible side-effects. Briefly, HEPES activated lysosomal transcription factors in macrophages (Tol et al., 2018), inhibited the prion protein conversion in neural stem cells and affected their viability and differentiation (Delmouly et al., 2011). Another study showed cellular uptake of HEPES in human cell lines (MCF-7, U2OS, HeLa) that persisted for 48 h after cells were returned to HEPES-free media (Depping and Seeger, 2019).

Recent assessments also shows that NVB addition may not fully prevent pH declines in standard batch cultures and may lead to unexpected pH changes when interacting with the HCO_3_^−^/CO_2_ buffering system, although predictable pH levels can be obtained when appropriate protocols are used (see, Michl et al., 2019). Researchers often manipulate medium pH by titrating acids and bases to achieve a desired level. The titration of acids and bases (including HCl, NaOH, and NVBs) introduces osmolytes (Na^+^, Cl^−^) to cell medium and can result in substantial changes to medium osmolarity by > 10% (Michl et al., 2019). Supra-physiological osmolarity can directly affect cell membrane tension and volume (Pedersen et al., 2013), but can also moderate how cells respond to other environmental parameters (Dezengotita et al., 1998). For instance, hybridoma cells exposed to elevated CO_2_ conditions exhibited reduced growth rates when osmolarity was held constant at 361 mOsm kg^−1^, but cell growth rates further declined by 30% when medium osmolality was 415 mOsm kg^−1^ (Dezengotita et al., 1998).

## The Solutions

### Measure Environmental Parameters

Key environmental parameters (temperature, O_2_, CO_2_, and pH) should be accurately measured and reported. Researchers should also consider measurements of osmolarity and hydrostatic pressure (if experiments are not conducted at atmospheric pressure) because these variables are required for unit conversions of dissolved gases (Christmas and Bassingthwaighte, 2017), thereby facilitating accurate replication and comparisons of conditions among studies. Ideally, measurements of key parameters (O_2_, CO_2_, and pH) should be conducted to capture the variability that cell cultures experience, either continuously where logging systems can be used or via non-autonomous, regular measurements. A basic understanding of the expected variability for each of these parameters in specific experimental setups can be used to help guide the frequency of measurements required to capture the variability. As a minimum requirement in routine cell cultures, initial and final values are required for cases of linear drift characteristic of many batch culture experiments (Michl et al., 2019), whereas frequent recording (e.g., 1-min intervals) are likely required for advanced bioreactor systems involving gas and/or acid and base additions. Measuring these parameters at concurrent time points is critical to understand the interdependencies among parameters, and guide the explanation of their possible forcing on cellular responses (e.g., proliferation, metabolism, changes in gene transcription, epigenetic regulation). For instance, the solubility of dissolved gases, and thus the influence of CO_2_ on acid-base chemistry, is strongly dependent on temperature, osmolarity, humidity, and pressure (Christmas and Bassingthwaighte, 2017). Although thermal regimes of cell cultures may be reliably inferred from calibrated incubators, levels of dissolved O_2_ and CO_2_, as well as pH must be measured directly in the culture medium because cellular metabolism directly affects these parameters (Supplementary Table S1). Measuring systems capable of delivering the required precision and accuracy are available for all key environmental parameters. Such systems range in cost, from moderately priced sensors for temperature, pH, O_2_, and salinity to the more expensive sensing equipment required for monitoring dissolved CO_2_, which often require complex calibration protocols (Supplementary Table S1).

Levels of relative humidity and media evaporation are equally important considerations for the control of cell culture environments. Since variations in both factors result in changes to osmolarity as well as solute and gas concentrations, that in turn, affect diffusion. Unfortunately, variations in relative humidity even in sophisticated incubators are common (Triaud et al., 2003), but they should be recognized and remedied. Low-cost sensors are available to monitor relative humidity levels inside incubators (Supplementary Table S1, Rajan et al., 2017) and should also be reported alongside key environmental variables. For relative humidity and the minimization of medium evaporation, water baths or pans placed inside incubators provide a simple and cost-effect solution to maintaining adequate levels, although this approach is limited in its capacity for precise control and can elevate the risk of condensation and contamination. Watertight joints in culture incubators may also require frequent maintenance and replacement, where needed (Rajan et al., 2017). More sophisticated and costly options are available for the control of relative humidity (and minimization of medium desiccation), including direct steam humidification systems and incubators capable of two-sided controls (Supplementary Table S1).

**FIGURE 1:**
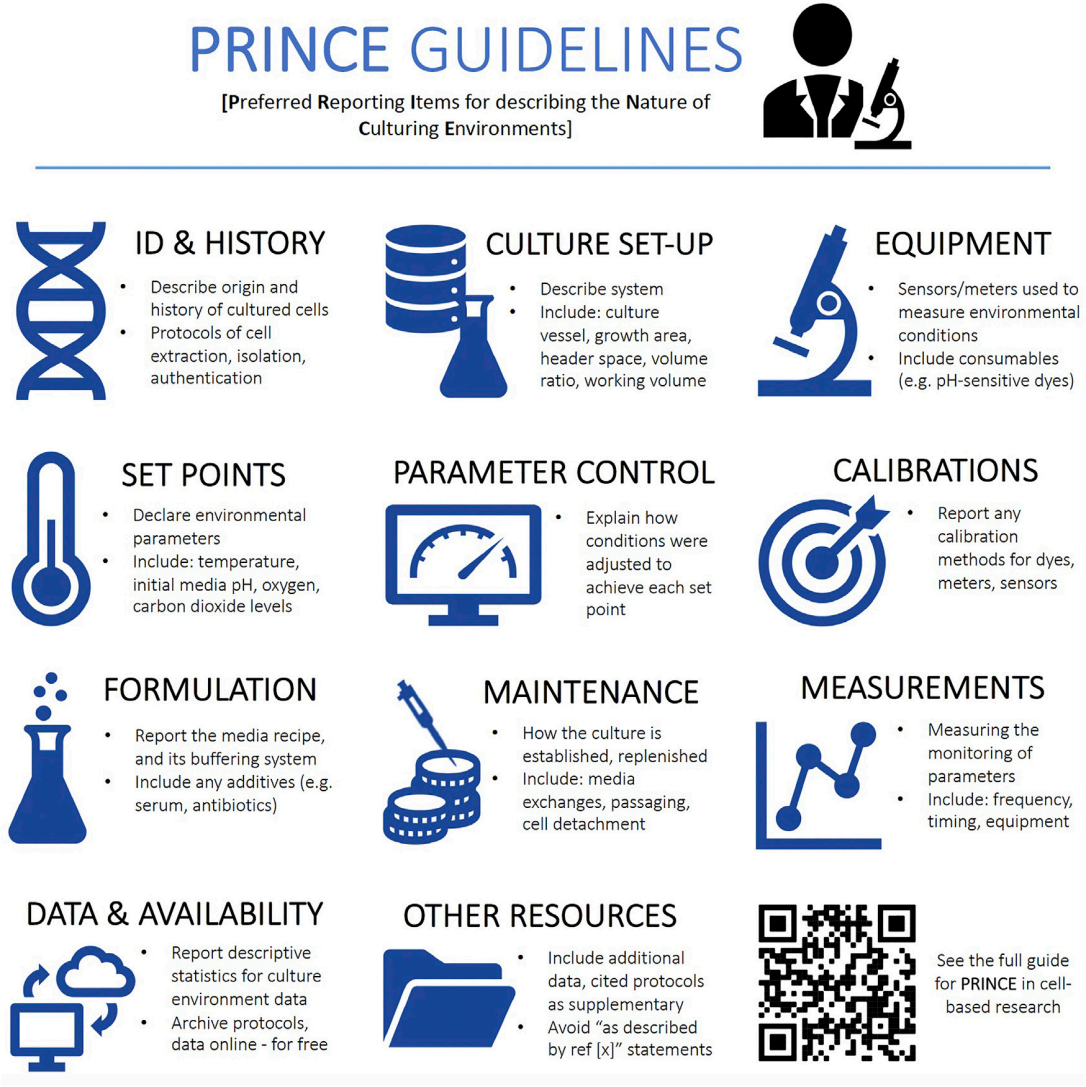
An overview of the PRINCE (Preferred Reporting Items for describing the Nature of Culturing Environments) guidelines. The QR provides access to the full PRINCE reporting checklist (also in Supplementary Extended Materials S1) containing an exhaustive list of reporting items.

Accordingly, we provide reporting guidelines (Figure 1, Supplementary Extended Materials S1) along with sample method descriptions (Supplementary Extended Materials S2), to guide practitioners into conducting and reporting characterizations of environmental regimes and promote a greater understanding of the factors that may affect precision and accuracy of experiments. Indeed, researchers could consider conducting pilot experiments to understand if variability in environmental factors significantly affects key experimental readouts.

### Control Environmental Parameters

**FIGURE 2:**
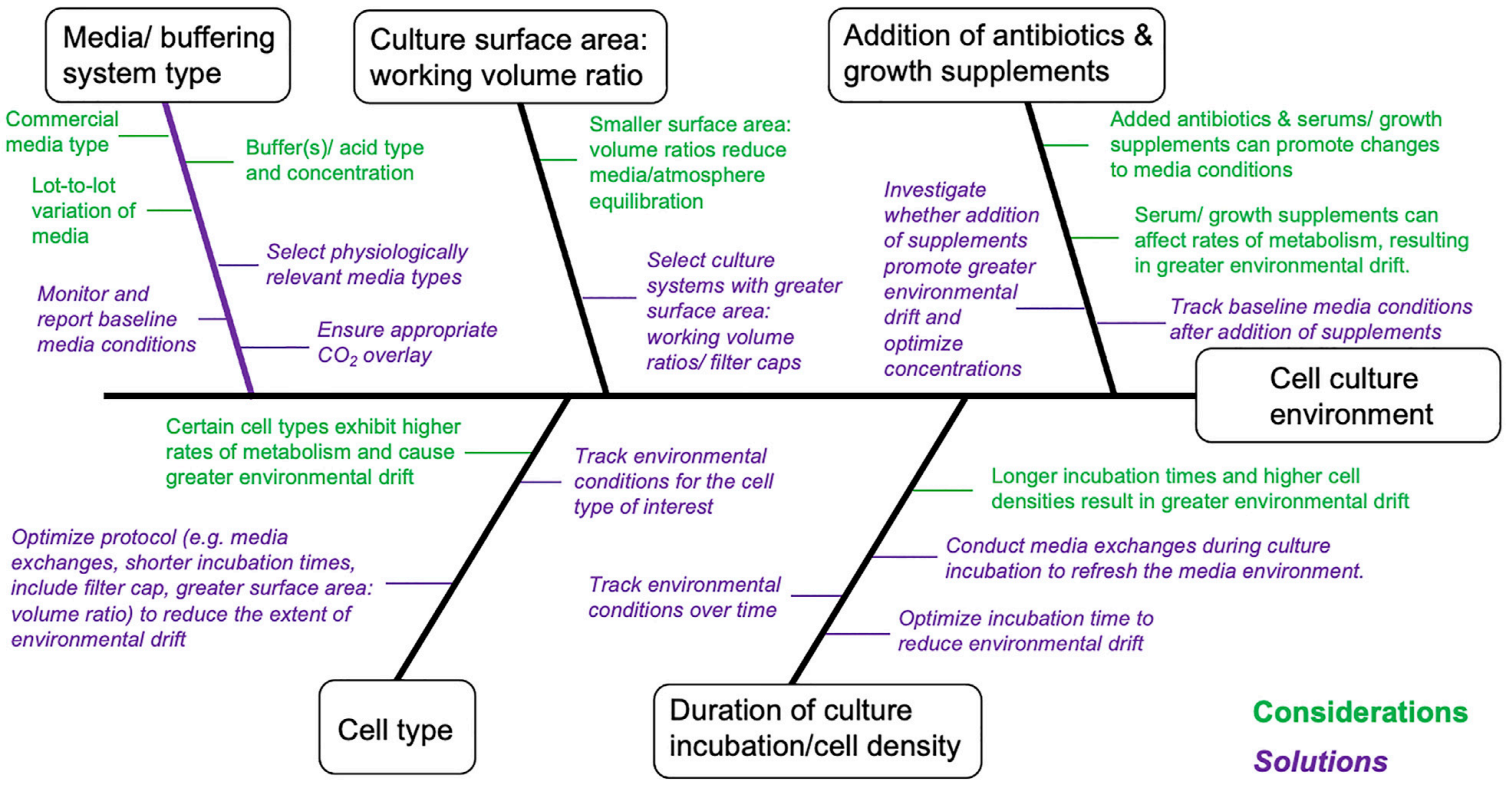
Cause-and-effect diagram summarizing considerations that can directly and indirectly affect environmental conditions in *in vitro* cell cultures. This diagram is non-exhaustive and should be further complemented/modified for specific cellular models and/or fields of research. Statements marked in green are technical considerations affecting *in vitro* cell cultures and statements marked in Purple (italicized) are proposed solutions.

A reasonable degree of control over environmental conditions is achievable in routine culture systems, but requires consideration of workflow factors that can, directly and indirectly, promote environmental drift (Figure 2). Interpretation and reproducibility of biomedical experiments involving mammalian cell cultures mandate that environmental parameters (temperature, O_2_, CO_2_, and pH) be at least monitored and reported, and where possible environmental variation minimized and controlled. Environmental stability is most easily achieved in advanced bioreactor culture systems, whereas achieving stability in routine batch culture systems is most challenging, with perfusion systems (and chemostats) providing intermediate solutions (Table 1). Batch cultures are typically maintained in incubators that maintain temperature (typically 37°C) and guarantee a CO_2_ level (typically 5%) in the atmosphere. In batch culture set-ups, researchers often select culture medium that contains a pH indicator dye [e.g., Phenol Red (PhR)] to guide the renewal of cell medium, but medium color changes assessed “by eye” can lead to undetected pH declines (Michl et al., 2019). Indeed, batch cell cultures are the most popular, inexpensive and scalable culture system, in terms of the possible number of replicates and treatments, with <1% of the published literature utilizing cybernetic bioreactor or chemostat culture systems (2014–2019, Klein et al., 2021b). Chemostat and perfusion systems were first described in 1950 for use in bacterial cultures (Novick and Szilard, 1950a; b) and later adopted for mammalian cells in 1961 (Cohen and Eagle, 1961). Such systems can maintain environmental conditions and cell growth rates via a continuous dilution of the culture with fresh medium (Cohen and Eagle, 1961). More sophisticated bioreactor culture systems were first introduced in the 1970s to culture mammalian cells (Knazek et al., 1972; Fouron, 1987) and typically involve the automated control of temperature, gas addition (O_2_, CO_2_), and/or acids and bases to maintain set targets for temperature, dissolved gases and pH. Bioreactors provide the best capacity to control environmental conditions, but are most costly in the context of capital investment, maintenance, and operations. Importantly, many bioreactor systems lack flexibility in the number of biological replicates and the volume of culture media (often larger than that of batch culture) that can be manipulated, which translates into greater time and monetary costs. While traditional bioreactor systems are ideal for cells culture in suspension, attached cell monolayers require different solutions. For these cell types, advanced bench top culture systems providing convection of culture media (Supplementary Table S1), which are also expandable in terms of replication, will likely provide the best capacity for adherent cell types (see, Kreß et al., 2021). We provide considerations as well as suggestions for improved environmental control for culture systems ranging in complexity from batch cultures to bioreactors (Table 1 and Supplementary Table S2).

**TABLE 1:** Constraints, advantages, and solutions for improved environmental control and reproducibility for three major types of culture systems.

Culture system	Constraints	Advantages	Solutions
Monitored Batch	Limited control of environmental conditions; limited reproducibility	Effective temperature control; affordability; low maintenance; high replication possible; sterilization and autoclaving of vessels not required	Monitor environmental conditions (optimize protocol to reduce environmental drift; report environmental conditions and detailed protocols
Chemo stat/Perfusion set-ups/Micro-fluidics	Time investment in optimizing set-up; moderate maintenance required; moderate cost for equipment; high consumption of consumables	Affordability; effective control of conditions; control of growth rates of suspended cells; small - moderate scale replication possible	Monitor environmental conditions; optimize flow/perfusion rates; report environmental conditions and detailed protocols
Bioreactor	High-cost; high consumption of consumables / typically require larger volumes of media	Precise control of environmental conditions; control growth rate of suspended cells; high-frequency environmental monitoring. Scalable in the number of culture vessels	Randomize and repeat experiments on small-scale bioreactor set-ups; report environmental regimes and detailed protocols

### Report Procedures to Monitor and Control Environmental Data

Precise control and monitoring of environmental conditions for mammalian cell cultures need be accompanied by reporting of the procedures used—a requirement, that is, not yet sufficiently emphasized nor enforced by the majority of scientific journals. A survey assessing the reporting requirements of leading biomedical journals for publications involving mammalian cells (Cell, Nature, Science, etc.; cf. Supplementary Extended Materials S3) revealed that only *Nature Research, Science, Cell,* and *EMBO press* journals require a standardized declaration of reporting practices to be published as a form attached to the electronic version of the published papers (see Nature Reporting Standards and MDAR Reporting Standards). However, none of the information required in this form addresses the monitoring and control of critical environmental conditions for research conducted using mammalian cell cultures (hereafter referred to as “cell-based experiments”). Strengthening reporting requirements and standards will likely place greater emphasis on cell culturing environments and, in turn, likely enhance the reproducibility of cell-based experiments as well as their relevance to the *in vivo* environment. We address this gap by offering the PRINCE (Preferred Reporting Information on the Nature of Cell-culturing Environments) guidelines (Supplementary Extended Materials S1) as a checklist for the parsimonious reporting of the monitoring and control of critical environmental conditions in the experiments reported in the papers. The PRINCE reporting checklist is designed to be adopted by journals publishing cell-based experiments and be included as a required declaration at the time of submission, thereby available to be assessed by peer reviewers, to then become available as an appendix to the electronic version of the published papers (Supplementary Extended Materials S1). This will ensure much needed standardized reporting of cell culture conditions.

### Report Resulting Environmental Data

Monitoring and controlling environmental conditions for cell-based experiments must be accompanied by reporting the data obtained as an essential step to identify possible environmental artifacts affecting the reproducibility of the findings and their comparisons among studies. The lack of detailed methodological and data reporting prevalent in studies published to date has been attributed to strict word and page limits enforced by publishers (Freedman et al., 2015). However, while many journals, dictate strict restrictions on the main body of the published text, most journals encourage providing all relevant details in extended materials, thereby extending the space available to accurately describe the procedures used and report additional data helping interpret the results presented. Online repositories are also available for more detailed reporting of protocols (e.g., Nature’s Protocol Exchange, Dryad) and datasets (e.g., Dryad, figshare and Zenodo). These data repositories were designed to meet journal and funder requirements for data availability and most of these offer data curation services that streamline the uploading process and ensure sustained access to the data.

Minimum reporting requirements should include the mean and a metric of dispersion (e.g., SD, SEM or range) for each of the monitored environmental parameters. In cases where environmental parameters display a monotonous trend over time, the slope, a metric of dispersion, as well as the probability of the slope being equal to zero may be reported to describe the change over time and can be fitted using simple linear regression analysis. Ideally, researchers would accompany such summary statistics with a supplementary figure displaying environmental regimes over time (Michl et al., 2019), so that the published findings can be interpreted alongside the nature of cell-culture conditions.

### Recommendations

The task of enhancing standards for environmental control, monitoring, and reporting in biomedical research may initially seem overwhelming provided the current absence of a culture to this end (Collins and Tabak, 2014; Baker, 2016; Hunter, 2017). Enhancing standards is also hindered by the limited availability of affordable culture systems capable of advanced environmental monitoring and conrtol for a broad range of cell types. The lack of appropriate tools in turn contributes to the lack of awareness of the true extent of environmental instability. However, reporting only nominal set-points used in cell culture systems without verification cannot be a sustainable solution.

**FIGURE 3:**
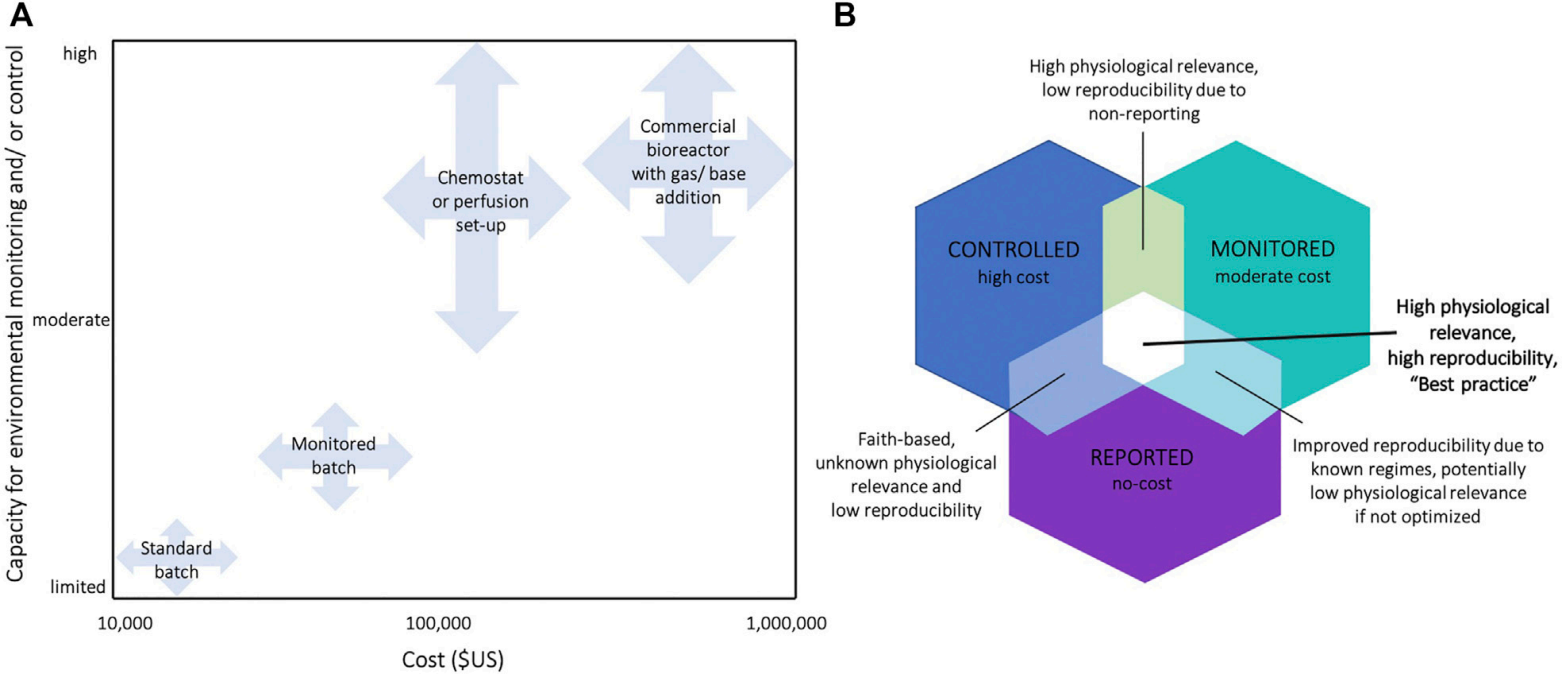
**(A)** Classification of cell culture systems by their capacity for environmental control/and or monitoring, and estimates of the required financial investments. Estimates for the required financial investment include the full costs associated with the required set-ups, including the key infrastructure required (see Supplementary Table S2 for prices of exemplary commercial systems) **(B)** Venn diagram summarizing the benefits and disadvantages of monitoring, controlling, and/or reporting cell culture environments in experiments. E.g., acquirement of a bioreactor is high in cost, but often only setpoints are reported without actually measuring environmental conditions in the medium (mean ± SE) leading to unknown physiological relevance and low reproducibility.

Resolving the issue without a systematic approach may risk putting more burden on researchers’ time, resources, and expertise. As an immediate requirement, initial and final values of key parameters should be measured and reported in the batch cultures of cell lines used in experiments. This requirement should capture existing environmental variation affecting published findings, ensuring accurate interpretation of the reported results and improved reproducibility. The provided PRINCE reporting checklist is designed to apply to a range of culture systems, from routine batch cultures to advanced culture systems (e.g., prefusion set-ups and bioreactors). Next, existing protocols must be optimized to minimize environmental variation in routine cultures (Supplementary Table S1). The third step is to build the capacity and infrastructure, supported by a sufficient understanding of the causes and consequences of variability in these conditions. Where needed, postgraduate biomedical programs may be revised to strengthen these competences. The next step, which requires significant investment over longer time frames (years to decades), involves the routine use of advanced cell culture technologies that allow precise and accurate control and monitoring of environmental conditions (Figure 3, Supplementary Table S1). Pending these advancements, reporting requirements should then extend to include proliferative, maintenance culture vessels, not only those dedicated to experimental assessments. Researchers should then consider the relevance *in vitro* culture environments to the levels under which particular cell types exist *in vivo*. For instance, O_2_ levels vary across human tissues and range from 13% O_2_ in the lung-pulmonary vein to 1–3% O_2_ in the uterus (Ast and Mootha, 2019). By considering how niche *in vivo* environments affect experimental outcomes, researchers could further increase the robustness of their experiments and increase the likelihood that findings have relevance to focal *in vivo* compartments (Ast and Mootha, 2019). Particular fields within biomedical science (e.g., 3D cultures and stem cell research) are already making great strides in this arena (Ryall et al., 2015; Shyh-Chang and Ng, 2017), although reliable reports of environmental parameters within selected human tissues are presently limited (Al-Ani et al., 2018; Ast and Mootha, 2019) and this research area warrants further attention.

Ultimately, improved characterization and control over environmental conditions in cell cultures will enhance the reliability of experimental findings and the confidence in their translation to clinical applications, which should provide sufficient rationale for funding bodies and institutions to invest in the necessary infrastructure. Indeed, funding agencies could consider supporting research initiatives aiming to further investigate the effects on environmental factors on commonly studies biological responses (i.e., gene expression, histone modification, metabolic pathways) in model cell lines (e.g., Bumke et al., 2003; Ben-David et al., 2018; Muelas et al., 2018). This will provide a systematic understanding of the impacts of environmental control on cell culture experiments. Transitioning towards advanced culture systems capable of mimicking *in vivo* conditions not only requires consideration of environmental parameters, but also necessitates attention to other chemical and physical factors known to program cell fate. Such factors include, but are not limited to, the common usage non-physiological concentrations of growth factors (Rubin, 2007; Holohan et al., 2013; Langhans, 2018), and antibiotics (Ryu et al., 2017), as well as the physical structure of cell microenvironments, which can alter cell morphology and function (Darnell et al., 2018). For many of these factors, existing recommendations that aim to increase the relevance of *in vitro* cultures to *in vivo* physiology are available (Baker, 2016; Muelas et al., 2018; Hirsch and Schildknecht, 2019).
