# Supplementary material for: Toward Best Practices for Controlling Mammalian Cell Culture Environments
Source: Front Cell Dev Biol. 2022 Feb 21;10:788808. doi: 10.3389/fcell.2022.788808 (PMC8900666; doi:10.3389/fcell.2022.788808)
Supplement: Supplementary file 1 [file Table1.docx]

**Table S1.** Challenges and solutions for improved environmental monitoring of temperature, pH, dissolved oxygen (O_2_), and dissolved carbon dioxide (CO_2_) live-cell culture systems.

| **Parameter** | **Challenges** | **Advantages** | **Solutions for improved environmental monitoring** |
| --- | --- | --- | --- |
| **Temperature** | Commercially available thermometers can give inaccurate readings (± 1 ^o^C error). | A stable thermal environment ensures accurate control of dissolved gases and acid-base chemistry.  Low cost, but accurate measurements. | Traceable thermometers and temperature logging systems calibrated by an ISO [International Standards Organization] certified laboratory ensure accurate and precise temperature readings. ISO-certified thermometers are widely available at low cost (< US $100). |
| **pH/ acid-base chemistry** | Changes in media pH are commonly assessed using pH indicator dye. However, media color assessments that are done “by eye” may produce inaccurate readings. | Simple to measure with electrodes or *indicator dyes*.  Cost-effective and accurate measurements. | pH sensors calibrated with traceable buffer solutions (e.g. primary standards set by NIST [National Institute of Standards and Technology]) ensure highly accurate and precise pH readings.  pH meters and sensors are widely available at low to moderate costs (between US $300 -$1000). Traceable standard buffer solutions available at low cost (< US $100). Sensors compatible with sterilization and autoclaving are available.  pH sensing technologies are available for non-invasive measurements in cell cultures.  Color assessments of pH indicator dyes via absorbance spectrum analysis provide accurate measurements when calibrated with traceable standard buffer solutions. |
| **Dissolved O_2_** | Gaseous calibrations may be required (typically a two-point calibration of 100% air saturation, and 0% O_2_). | Accurate tracking of O_2_ regimes (e.g. hypoxic exposure).  Cost-effective and accurate measurements. | Dissolved O_2_ meters and sensors are widely available at low to moderate costs (between US $400- $2000). Dissolved O_2_ meters that compensate for temperature, altitude (pressure), and salinity are available. Dissolved O_2_ sensors compatible with sterilization and autoclaving are available.  Dissolved O_2_ sensing technologies are available for non-invasive measurements in cell cultures.  Simple calibration protocols for most sensors, typically involving air and N_2_ gas (100% saturation, and 0% O_2_, respectively). |
| **Dissolved CO_2_** | High-cost sensing systems.  Manufacturer calibrations may be required. | Permits tracing of CO_2_ regimes in culture, independent of pH (and acid-base regulation). | CO_2_ meters and sensors can achieve accurate and precise CO_2_ readings. Dissolved CO_2_ sensors compatible with sterilization and autoclaving are available.  Dissolved CO_2_ sensing technologies are available for non-invasive measurements in cell cultures, but some commercially available technologies are in the prototype phase. |
| **Agitation** | Agitation rates (e.g. impeller speed in bioreactor set-ups) control shear force affecting cell experimental responses. | Stable rates of agitation ensure accurate control of gas/atmosphere equilibration and uniform environmental conditions (i.e. prevent gradient from the air-medium interface to the cell layer). | Report rates of agitation/ flow. |
| **Relative humidity** | Variations in relative humidity are common.  Affects osmolarity as well as solute and gas concentrations, that in turn, affect diffusion. | Adequate levels and control of humidity prevent changes in medium viscosity, concentrations of salts and ions, gas solubility and osmolarity. | Low-cost sensors are available to monitor relative humidity levels inside incubators. Thermoset polymer based capacitive humidity sensors are fast, linear and are stable long-term. They also compatible with internal incubator conditions and available for in-line set-ups.  Advanced control systems for relative humidity are also available, including direct steam humidification and bidirectional humidity control. |
