# Supplementary material for: Toward Best Practices for Controlling Mammalian Cell Culture Environments
Source: Front Cell Dev Biol. 2022 Feb 21;10:788808. doi: 10.3389/fcell.2022.788808 (PMC8900666; doi:10.3389/fcell.2022.788808)
Supplement: Supplementary file 2 [file Presentation1.pdf]

BOX 1

Role of metabolic gases (O_2 and CO_2) in mammalian fluids and cell culture media

Homeostasis describes the maintenance of a steady-state in an organism. In mammalian bodies, this includes maintaining the equilibrium of temperature, water (H_2O), physiological processes involving oxygen (O_2), carbon dioxide (CO_2), and pH of extracellular fluids, but also blood sugar levels, and the concentrations of sodium-, potassium- and calcium ions. Acid-base chemistry and levels of dissolved gases within mammalian fluids are driven by aerobic and anaerobic metabolic processes (Eq. 1 – 2), and regulated by the respiratory and cardiovascular systems.

Aerobic metabolism

$C_6H_{12}O_6$ + 6 O_2 → 6 CO_2 + 6 H_2O + energy (30-32 ATP molecules + heat) Eq. 1

Anaerobic metabolism

$C_6H_{12}O_6$ → 2 Lactic Acid + 2 ATPs Eq. 2

The critical role of dissolved gases in moderating physiological processes has long-been recognized [1], but gained newfound attraction upon the discovery that cells can sense and adapt to varying levels of O_2, revealing oxygen as a primary regulator of fundamental physiological processes (2019 Nobel Prize in Physiology or Medicine). O_2 and CO_2 are important signalling molecules but fundamentally differ in their behaviour upon dissolution in metazoan fluids and culture media. CO_2 dissociates to produce carbonic acid and spontaneously forms H^+ ions and reacts with HCO_3^- to stabilize pH in the body (Eq. 3-4).

pCO_2(aq) + H_2O(aq) ⇌ H_2CO_3(aq) ⇌ HCO_3^-(aq) + H^+(aq) Eq. 3

Anaerobic metabolism influences media pH

Lactic acid ⇌ Lactate + H^+ Eq. 4

In cell culture medium, the bicarbonate buffering system operates through the Le Chatelier's principle (Eq. 5). Commercial incubators with a CO_2-rich atmosphere (usually set to 5%) enable the CO_2/HCO_3^- buffering. Free carbonic ions in the medium react with the extra H^+ ions to form carbonic acid, and stabilize pH.

CO_2(gas) ⇌ HCO_3^-(aq) + H^+(aq) Eq. 5

Ideal Gas Law

PV = nRT Eq. 6

where P = pressure (mmHg), V = volume (L), n = number of molecules of gas (mol), R = ideal gas constant (62.364 L mmHg K−1 mol−1), and T = temperature (K). [3]

Henry's Law states that dissolved oxygen (µM) in the liquid medium is proportional to the partial pressure of oxygen in air. In solution, oxygen solubility decreases with increasing temperature and ionic strength and increases with atmospheric pressure.[4]

Henry's Law

$$P \cdot \gamma_{O_2} = H \cdot x^* \qquad \text{Eq. 7}$$

where P is pressure of the gas mixture above the medium, γ_{O_2} is the mole fraction of O_2 within this mixture, H is Henry's constant, and x^* is the equilibrium mole fraction of O_2 in the medium immediately adjacent to the interface.[5]

Henderson-Hasselbalch equation

pH = pK_a + log([B]/[HB]) Eq. 8

where pH is related to the acid dissociation constant (pK_a) and concentration of the buffer's unprotonated [B] and protonated [HB] forms. [2]

Within the human body, finite limits of O_2, CO_2, and pH differ between organs and fluids. O_2 and pH follow a reverse trend than the finite limits of CO_2 [6].

In vivo **homeostasis and finite limits (*e.g.*, Arterial blood gas [7,8]) versus *In vitro* variability [9]**

Variable	*In vivo*	*In vitro*
pH	7.35 to 7.45	6.65 to 7.4
pCO_2	35 to 45 mmHg	23 to 137 mmHg
pO_2	75 to 100 mmHg	118 to 167 mmHg
HCO_3^-	22 to 26 mEq	-

As shown in Human erythroleukemia cells (K562), *in vitro* values can drift as much as to a pH 6.7, CO_2 11% and O_2 16%, which is outside *in vivo* finite limits [9].

In the human body, respiratory and cardiovascular systems (among other systems and mechanisms; e.g., kidneys) ensure that cells *in vivo* receive sufficient O_2 and acid-base stability. These systems are in charge of pH homeostasis, and would be difficult to maintain withouth acid urinary excretion, and the ability to generate new bicarbonate. Failure to regulate dissolved gases and acid-base chemistry within the finite limits that maintain homeostasis [10] can be hallmarks of numerous hormone and metabolic disorders, as well as carcinogenesis [11].

List of References:

1 Behnke, A. R., Shaw, L. A., Shilling, C. W., Thomson, R. M., & Messer, A. C. (1933). Studies on the effect of high oxygen pressure: I. Effect of High Oxygen Pressure upon the Carbon-Dioxide and Oxygen Content, the Acidity, and the Carbon-Dioxide Combining Power of the Blood. *American Journal of Physiology-Legacy Content*, 107(1), 13-28.

2 Michl, J., Chan Park., K., & Swietach, P. (2019). Evidence-based guidelines for controlling pH in mammalian live-cell culture systems. *Communications Biology*, 2, 144.

3 Place, T. L., Domann, F. E., & Case, A. J. (2017). Limitations of oxygen delivery to cells in culture: An underappreciated problem in basic and translational research. *Free Radical Biology and Medicine*, 113, 311-322.

4 Ast, T., & Mootha, V. K. (2019). Oxygen and mammalian cell culture: are we repeating the experiment of Dr.Ox? *Nature Metabolism*, 1, 858–860.

5 Al-Ani, A., Toms, D., Kondro, D., Thundathil, J., Yu, Y., & Ungrin, M. (2018). Oxygenation in cell culture: Critical parameters for reproducibility are routinely not reported. *PLoS One*, 13(10), p.e0204269.

6 Keely, T. P., & Mann, G. E. (2019). Defining physiological normoxia for improved translation of cell physiology to animal models and humans. *Physiol Rev*, 99, 161–234.

7 Hopkins, E., & Sharma, S. (2019). Physiology, acid base balance. [Updated 2019 Jun 16]. *In: StatPearls [Internet]*. Treasure Island (FL): StatPearls Publishing.

8 Thews, G. (1989). Pulmonary Respiration. In: Human Physiology, Eds: Schmidt and Thews. Springer-Verlag Berlin Heidelberg, Germany, pp. 825.

9 Klein, S. G., Alsolami, S. M., Arossa, A., Ramos Mandujano, G., Parry, A. J., Steckbauer, A., Duarte, C. M., & Li, M. (2022). In situ monitoring reveals cellular environmental instabilities in human pluripotent stem cell culture. Accepted to *Communications Biology*.

10 Keyes, J. L. (1990). Fluid, Electrolyte, and Acid-Base Regulation. Jones & Bartlett Publishers Inc.

11 Hanahan, D., & Weinberg, R. A. (2011). Hallmarks of cancer: the next generation. *Cell*, 144(5), 646-74. doi: 10.1016/j.cell.2011.02.013. PMID: 21376230
