# Supplementary material for: Toward Best Practices for Controlling Mammalian Cell Culture Environments
Source: Front Cell Dev Biol. 2022 Feb 21;10:788808. doi: 10.3389/fcell.2022.788808 (PMC8900666; doi:10.3389/fcell.2022.788808)
Supplement: Supplementary file 4 [file Table2.docx]

**Table S2.** Examples of advanced commercial culture systems, including perfusion-based systems and expandable bioreactor set-ups.

| Company name | Product | System type | Cell culture type | Estimated price of complete set-up (USD) | Estimated time for set-up/optimization | Publications |
| --- | --- | --- | --- | --- | --- | --- |
| Ibidi | ibidi Pump System Quad | perfusion system | adherent | 20,000 – 30,000 | 1-3 months | Del Favero et al., 2018 |
| Ibidi | ibidi Pump System | perfusion system | adherent | 17,000 – 25,000 | 3-5 months | Qu et al., 2020 |
| Ske | CC100 InFlow bioreactor | bioreactor | adherent 3D with scaffold | 50,000 – 60,000 | 3-5 months | - |
| Ske | CC200 InFlow-R bioreactor | bioreactor | adherent with scaffold | 65,000 –75,000 | 3-5 months | - |
| Eppendorf | DASGIP® Parallel Bioreactor Systems | Bioreactor (expandable) | suspension | 100,000 - 600,000 | 3-5 months | Kim et al., 2012  Agabalyan et al., 2017 |
| PALL Biotech | Allegro™ XRS 25 Bioreactor System | bioreactor | suspension | 40,000 - 90,000 | 3-5 months | Hunter et al., 2019 |
| PALL Biotech | Allegro™ STR Single-use Stirred Tank Bioreactors | bioreactor | suspension | 40,000 - 90,000 | 3-5 months | Schirmer et al., 2020 |
| PALL Biotech | iCELLis® Single-Use Fixed-Bed Bioreactor Systems | bioreactor | adherent | 40,000 - 90,000 | 3-5 months | Lesch et al., 2015 |
| PALL Biotech | Xpansion® Multiplate Bioreactor System | bioreactor | adherent | 40,000 - 90,000 | 3-5 months | Lambrechts et al., 2016 |
| Esco Lifesciences GmbH, Germany | CelCradle™ | bioreactor | adherent | 25,000 - 50,000 | 3-5 months | Rhazi et al., 2021 |
| regenerative-medtech | 3D Perfusion Unit | perfusion system | adherent | 20,000 - 40,000 | 3-5 months | - |
| amsbio | Reinnervate Perfusion Plate | perfusion system | adherent | 20,000 - 40,000 | 3-5 months | - |
| Reprocell | Alvetex™ Perfusion Plate and Alvetex Scaffold 6 Well Inserts Kit | perfusion system | adherent | 20,000 - 40,000 | 3-5 months | - |
| Reprocell | Alvetex™ Scaffold Multiwell Plates | perfusion system | adherent | 20,000 - 40,000 | 3-5 months | - |

References

Agabalyan, N.A., Borys, B.S., Sparks, H.D., Boon, K., Raharjo, E.W., Abbasi, S., Kallos, M.S. and Biernaskie, J., 2017. Enhanced expansion and sustained inductive function of skin‐derived precursor cells in computer‐controlled stirred suspension bioreactors. Stem cells translational medicine, 6(2), pp.434-443.

Del Favero, G., Zaharescu, R. and Marko, D., 2018. Functional impairment triggered by altertoxin II (ATXII) in intestinal cells in vitro: cross-talk between cytotoxicity and mechanotransduction. Archives of toxicology, 92(12), pp.3535-3547.

Hunter, M., Yuan, P., Vavilala, D. and Fox, M., 2019. Optimization of protein expression in mammalian cells. Current protocols in protein science, 95(1), p.e77.

Kim, B.J., Diao, J. and Shuler, M.L., 2012. Mini‐scale bioprocessing systems for highly parallel animal cell cultures. Biotechnology progress, 28(3), pp.595-607.

Lambrechts, T., Papantoniou, I., Viazzi, S., Bovy, T., Schrooten, J., Luyten, F.P. and Aerts, J.M., 2016. Evaluation of a monitored multiplate bioreactor for large-scale expansion of human periosteum derived stem cells for bone tissue engineering applications. Biochemical engineering journal, 108, pp.58-68.

Lesch, H.P., Heikkilä, K.M., Lipponen, E.M., Valonen, P., Müller, A., Räsänen, E., Tuunanen, T., Hassinen, M.M., Parker, N., Karhinen, M. and Shaw, R., 2015. Process development of adenoviral vector production in fixed bed bioreactor: from bench to commercial scale. Human gene therapy, 26(8), pp.560-571.

Qu, D., Wang, L., Huo, M., Song, W., Lau, C.W., Xu, J., Xu, A., Yao, X., Chiu, J.J., Tian, X.Y. and Huang, Y., 2020. Focal TLR4 activation mediates disturbed flow-induced endothelial inflammation. Cardiovascular research, 116(1), pp.226-236.

Rhazi, H., Safini, N., Mikou, K., Alhyane, M., Tadlaoui, K.O., Lin, X., Venkatesan, N.P. and Elharrak, M., 2021. Production of small ruminant morbillivirus, rift valley fever virus and lumpy skin disease virus in CelCradle™-500A bioreactors. BMC Veterinary Research, 17(1), pp.1-9.

Schirmer, C., Müller, J., Steffen, N., Werner, S., Eibl, R. and Eibl, D., 2020. How to produce mAbs in a cube-shaped stirred single-use bioreactor at 200 L scale. In Animal Cell Biotechnology (pp. 169-186). Humana, New York, NY.
