# Supplementary material for: Toward Best Practices for Controlling Mammalian Cell Culture Environments
Source: Front Cell Dev Biol. 2022 Feb 21;10:788808. doi: 10.3389/fcell.2022.788808 (PMC8900666; doi:10.3389/fcell.2022.788808)
Supplement: Supplementary file 5 [file DataSheet2.docx]

## Extended Materials 2

## Toward best practices for controlling mammalian cell culture environments *Shannon G. Klein^1^, Alexandra Steckbauer^1^, Samhan M. Alsolami^2^, Silvia Arossa^1^, Anieka J. Parry^1^, Mo Li^2*^, and Carlos M. Duarte^1*^*

1. Red Sea Research Centre (RSRC) and Computational Bioscience Research Center (CBRC), King Abdullah University of Science and Technology, Thuwal 23955, Saudi Arabia

2. Biological and Environmental Science and Engineering Division (BESE), King Abdullah University of Science and Technology, Thuwal 23955, Saudi Arabia

Corresponding author* Email: [carlos.duarte@kaust.edu.sa](mailto:carlos.duarte@kaust.edu.sa) [mo.li@kaust.edu.sa](mailto:mo.li@kaust.edu.sa)

###

### Robust methods for environmental parameter monitoring in cell culture experiments

Here, we provide sample methods of environmental parameter monitoring in two example culture systems, ranging in complexity. The first method applies to batch flask culture systems, where the medium environment is monitored for media pH, dissolved O_2_, and dissolved CO_2_. The second method describes the cybernetic control of medium conditions using a commercially available bioreactor system and includes the characterization of environmental parameters. The sample methods reported here are intended only as examples and have not been executed in a laboratory setting, nor are they intended as complete methods descriptions and thus do not include all PRINCE reporting items (e.g. reporting of results/ data availability).

Method 1: Measurements of dissolved oxygen, carbon dioxide, and pH in batch cell cultures using non-invasive sensor spot technology

Human chronic myelogenous leukemia cells (cell line K562, ATCC) were maintained in RPMI-1640 medium, supplemented with 15% FBS and 22 mM NaHCO_3_^-^, which employed the HCO_3_^-^/CO_2_ buffer system to maintain physiological pH. Cells were suspended at 200 k mL^-1^ and cultured in T-75cm^2^ flasks (in 20 mL working volume) with vented caps (see Table S1 for key resources). Cells were maintained in a Heracell^TM^ i150 incubator with the temperature set to 37 ºC with an atmosphere of 5% CO_2_ in air and humidity at 90%. The incubator was calibrated for temperature and CO_2_ injection one week prior to the commencement of the experiment. The K562 cell line was received without a passage number from ATCC. K562 cells were passaged three times (at 72-hour intervals) prior to the experiment.

Dissolved O_2_ (*d*O_2_), dissolved CO_2_ (*d*CO_2_), and pH sensor spots were used to monitor *d*O_2_, *d*CO_2_, and pH conditions in four batch culture flasks during a 72-hour passage. DO sensor spots were calibrated using a two-point calibration (100% air saturation, and 0% O_2_ via N_2_ gas [99.99% purity] bubbling), whereas the pH sensor spots were calibrated using a three-point calibration (using NIST traceable buffer solutions of pH 4, 7, and 10). Multi-channel fiber optic pH and *d*O_2_ meters were used to non-invasively measure *d*O_2_ and pH values from the sensor spots through the plastic wall of the culture flasks. Likewise, a single-channel fiber-optic *d*CO_2_ meter was used to non-invasively measure *d*CO_2_ values from the sensor spots through the plastic wall of the culture flasks. The sensor spots function as optodes, which upon activation by LED, act as an excitation light source and a photodiode to render *d*O_2_, *d*CO_2_, and pH measurements.

Three sensor spots (*d*O_2_, *d*CO_2_, and pH) were glued to the inside of each of the four T-75cm^2^ flasks using KwikSil silicone glue so that both the *d*O_2_, *d*CO_2_, and pH sensor spots were submerged in cell media in the horizontal position. All equipment used to install the sensor spots were autoclaved at 120 ºC for 20 minutes to prevent contamination. The flasks were left in darkness until the glue had curated (~18 min) to avoid any light-induced damage to the sensor spots and then each flask was sterilized using 50% EtOH, rinsed with DPBS, followed by RPMI-1640 media. Cells were inoculated at 200 k mL^-1^ in a 20 mL working volume of RPMI-1640 within each flask and placed under identical incubator conditions, as detailed above. pH and DO measurements were taken at the start of the experiment and then at 24-hour intervals over 72 hours (i.e., four time-points in total). Given previous observations of environmental drift for K562 cell cultures show a linear decline in *d*O_2_, *d*CO_2_, and pH, finer temporal scale measurements were considered unnecessary. The incubator door was opened for <1 min at each time-point so that measurements could be taken. At each time-point, the order of the flasks measured was rotated to minimize the influence of atmospheric CO_2_ depletion on the measurements, which occurs when the incubator atmosphere is interrupted over time. At the end of the experiment, cells were counted to ensure that each replicate flask achieved confluency. Specifically, the cell media was centrifuged (5 min at 250 rpm), and the cells resuspended in 5 mL of RPMI-1640 medium. A 20 µL aliquot was mixed with 20 µL of trypan blue for 3 min and counted using a Countess II automated cell counter. All culture flasks were then checked for mycoplasma contamination using Mycoalert kit and visibly checked for any sign of microbial contamination (i.e. bacteria).

**Table S1:** Key Resources Table for Method 1

| **REAGENT or RESOURCE** | **SOURCE** | **IDENTIFIER** |
| --- | --- | --- |
| **Reagents** |  |  |
| RPMI-1640 | Thermo Scientific™ | 31800105 |
| FBS | Gibco | 26140-079 |
| DPBS | Gibco | 14190144 |
| NIST traceable buffer solutions (pH 4.01) | Orion™ | ORIO910104 |
| NIST traceable buffer solutions (pH 7) | Orion™ | ORIO910760 |
| NIST traceable buffer solutions (pH 10.01) | Orion™ | ORIO9110CB |
| Sodium bicarbonate (NaHCO_3_^-^) | Gibco | 25080-102 |
| **Equipment** |  |  |
| T-75cm^2^ flasks (with vented caps) | VWR | 734-0050 |
| Heracell^TM^ i150 CO_2_ incubator | Thermo Scientific™ | 50116047 |
| Multi-channel fiber optic dissolved O_2_ meter | PreSens Precision Sensing GmbH | OXY-2 SMA |
| Single-channel dissolved CO_2_ meter | PreSens Precision Sensing GmbH | CO_2_-1 SMA |
| Multi-channel fiber optic pH Meter | PreSens Precision Sensing GmbH | pH-4 mini |
| Planar *p*CO_2_ mini sensor spots | PreSens Precision Sensing GmbH | SP-CD1-D5-rMy-US |
| Planar O_2_-sensitive spots | PreSens Precision Sensing GmbH | SP-PSt3-NAU-D5-YOP |
| Self-adhesive pH sensor spots | PreSens Precision Sensing GmbH | SP-LG1-SA |
| KwikSil silicone glue | PreSens Precision Sensing GmbH | 200001211 |
| Countess II automated cell counter | Thermo Scientific™ | AMQAF1000 |
| Mycoalert Kit | Lonza | LT07-118 |
| **Software** |  |  |
| PreSens Measurement Studio 2 version 3.0.3 | PreSens Precision Sensing GmbH | N/A |
| **Cell lines** |  |  |
| K562 | ATCC | N/A |

Method 2: Cybernetic control of temperature, dissolved oxygen, and pH conditions in a commercial bioreactor system

Human lymphoblastoid cells (cell line GM12878; Coriell Institute, received at passage 1) were cultured in RPMI-1640 medium supplemented with 10% FBS and 22 mM NaHCO_3_^-^ which used the HCO_3_^-^/CO_2_ buffer system. Cells were maintained in four culture vessels controlled by a commercial bioreactor system (Eppendorf DASbox Mini Bioreactor system controlled by DASware Software Suite). pH was maintained using a two-sided control via CO_2_ [99.99% purity] and NaHCO_3_^-^ buffer addition and O_2_ was maintained via a two-sided control using O_2_ and N_2_ gas [at 99.99% purity] addition. The bioreactor set-points were at 37 °C, 85% O_2_ saturation, and pH 7.4. Each vessel was equipped with pH, DO, and temperature sensors (see Table S2 for key resources) connected to (and controlled by) the bioreactor system software. Cells were checked daily for mycoplasma contamination using the Mycoalert kit and monitored for any sign of microbial contamination (i.e. bacteria).

Prior to the start of the experiment, pH sensors were calibrated via a two-point calibration (NIST traceable buffer solutions at pH 7 and 10). The bioreactor vessels were assembled, filled with DPBS, and subsequently autoclaved at 120 °C for 20 minutes. Post autoclaving, vessels were cooled at room temperature in a clean and pre-sterilized safety cabinet for ~1 hour. DO sensors were then calibrated in the DPBS solution using a two-point calibration (100 % air saturation, and 0 % O_2_ via N_2_ bubbling). The sensors were polarized overnight before the vessels were removed and externally sterilized with 70% EtOH. The DPBS solution was removed under the safety hood and 140 mL of RPMI-1640 medium was added to each vessel and the bioreactor system activated. Once pH and DO levels stabilized, cells were concentrated in 10 mL of RPMI-1640 medium and inoculated using an inoculation port, rendering a final concentration of 200 k cells mL^-1^ in 150 mL working volume. Temperature, DO, and pH levels were recorded every 30 min during the experiment and maintained mean levels of 37 ºC ± 0.001 (SD, *n* = 146), 89.82 % ± 4.12 % (SD, *n* = 146), and 7.397 ± 0.0129 (SD, *n* = 146), respectively.

**Table S2:** Key Resources Table for Method 2

| **REAGENT or RESOURCE** | **SOURCE** | **IDENTIFIER** |
| --- | --- | --- |
| **Reagents** |  |  |
| RPMI-1640 | Thermo Fisher | 31800105 |
| FBS | Gibco | 26140-079 |
| DPBS | Gibco | 14190144 |
| NIST traceable buffer solutions (pH 7) | Orion™ | ORIO910760 |
| NIST traceable buffer solutions (pH 10) | Orion™ | ORIO9110CB |
| Sodium bicarbonate (NaHCO_3_^-^) | Gibco | 25080-102 |
| **Equipment** |  |  |
| DASbox Mini Bioreactor | Eppendorf | OT30QUOTE |
| DASbox 250 mL vessel | Eppendorf | DS0250ODSS |
| pH Sensor | Hamilton® | 76DXPHHMC120 |
| DO Sensor | Hamilton® | 76DXPOHMC120 |
| 8-Blade impeller, 60°pitch | Eppendorf | 78107377 |
| Platinum RTS temperature sensor | Eppendorf | 78103308 |
| Mycoalert Kit | Lonza | LT07-118 |
| **Software** |  |  |
| DASware Software | Eppendorf | N/A |
| **Cell lines** |  |  |
| GM12878 | Corriel Insitute | N/A |
