# Supplementary material for: Toward Best Practices for Controlling Mammalian Cell Culture Environments
Source: Front Cell Dev Biol. 2022 Feb 21;10:788808. doi: 10.3389/fcell.2022.788808 (PMC8900666; doi:10.3389/fcell.2022.788808)
Supplement: Supplementary file 6 [file DataSheet1.docx]

**Extended Materials 1**

## Toward best practices for controlling mammalian cell culture environments *Shannon G. Klein^1^, Alexandra Steckbauer^1^, Samhan M. Alsolami^2^, Silvia Arossa^1^, Anieka J. Parry^1^, Mo Li^2*^, and Carlos M. Duarte^1*^*

1. Red Sea Research Centre (RSRC) and Computational Bioscience Research Center (CBRC), King Abdullah University of Science and Technology, Thuwal 23955, Saudi Arabia

2. Biological and Environmental Science and Engineering Division (BESE), King Abdullah University of Science and Technology, Thuwal 23955, Saudi Arabia

**Corresponding authors* |** Email: [mo.li@kaust.edu.sa](mailto:mo.li@kaust.edu.sa) [carlos.duarte@kaust.edu.sa](mailto:carlos.duarte@kaust.edu.sa)

**P**referred **R**eporting **I**tems describing the **N**ature of **C**ell-culturing **E**nvironments (**PRINCE**)

**Statement**: PRINCE is a set of preferred reporting items for scientific studies that involve mammalian cell cultures. PRINCE focuses on the reporting of cell-culturing environments with a particular focus on controlling and monitoring acid-base chemistry and dissolved gases, but can also be used as a basis for research investigating the role of environmental conditions in affecting mammalian physiological processes and mechanisms.

| **Reporting item checklist** | **item #** | **Description of preferred reporting item** | **Reported on page #** |
| --- | --- | --- | --- |
| **CULTURE SYSTEM** | | | |
| Cell-type | 1 | A statement describing the origin and history of cells cultured is included. Where applicable, a detailed method of primary cell extraction and isolation, and/or details of purchased cells is reported. For purchased cells, the cell-line name/identifier, source, passage number, authentication, and mycoplasma contamination are declared (see also [ICLAC](https://iclac.org/)). |  |
| Culture vessel type | 2 | The type of culture system (i.e., batch flask and plates, chemo-stat, or bioreactor control) is identified and described. Details of culture vessel attributes are reported, including the manufacturer, model, and working volume used. The closure type (e.g. filtered or sealed) and an estimate of the surface area: volume ratio is reported, regardless of the culture vessel used (e.g. culture flask, petri dish, tissue culture plate). |  |
| Media type | 3 | A detailed description of the media formulation is reported, including the manufacturer and product number, buffering composition (e.g., HCO_3_^-^/CO_2_ buffer-system), and supplements added (e.g., antibiotics, serum). If a bench-top adjustment of media pH was used, the method and the materials involved are declared. |  |
| Culture establishment and replenishment | 4 | A detailed description of how the culture system was established and maintained is included. Details may include seeding density, frequency of media exchanges, cell passaging, cell detachment, and cell differentiation, where applicable. If cells were embedded within extracellular matrix proteins, including, for example, collagen and laminin, or co-cultured with feeder cells, details were described. |  |
| **ENVIRONMENTAL CONTROL** | | | |
| Parameter(s) and set-points | 5 | The controlled parameters are identified and the set point for each parameter is reported (e.g. temperature was set at 37 °C). |  |
| Control approach | 6 | For each parameter, the method of control is reported and a description of how conditions were adjusted to achieve each of the desired set points is provided. Where applicable, the procedures by which gases and/or acids and bases were used to control parameters are clearly reported. Details describing the nature of control (e.g. addition/removal) and the use of feedback control systems (e.g. bioreactor equipped with sensors) are declared. |  |
| Equipment and consumables | 7 | Equipment and consumables used to control environmental conditions are described. Details of equipment may include, for example, incubator type, purchased bioreactor set-ups, and sensors, or peristaltic pumps. Details of consumables may include, type and purity of gases, or type and concentration of acids and bases. |  |
| **ENVIRONMENTAL MONITORING** | | | |
| Parameter(s) and method of measurement | 8 | All parameters measured are declared. A description of how each parameter was monitored in the culture system is reported, including the frequency and timing of measurements. Where appropriate, relative humidity of the incubator atmosphere was measured and reported. |  |
| Equipment and consumables | 9 | All equipment (e.g. meters and sensors) and consumables (e.g. pH-sensitive dye) used to monitor environmental conditions are identified and described. The description of meters and sensors includes details of the manufacturer and product code. |  |
| Identify units/scales of measurements | 10 | The unit of measurement for each parameter measured (e.g., mmHg, absolute %, % air saturation, ppm, μatm) is reported. The pH scale (*) used (NBS/NIST, total-scale, or free-scale) is reported in the manuscript. If more than one pH scale is used, pH values are reported with the associated scale as a subscript (NBS or NIST = pH_NBS or NIST_, total-scale = pH_T_, free-scale = pH_F_ (**)). |  |
| Calibration, accuracy, and sterilization | 11 | The method of calibration for individual meters/sensors or dyes is reported. Where applicable, details of the sterilization method is declared (e.g. autoclaved sensor). |  |
| **DATA REPORTING AND CITED MATERIALS** | | | |
| Data reporting | 12 | For all environmental parameters reported, mean levels, associated variance estimates (e.g., range, standard error, and standard deviation), and sample sizes (*n*) are reported and capture the extent of change experienced in the live-cell culturing system. Where possible, data has been summarized and/or displayed in a manner that permits an assessment of variability in cell culture conditions over time. |  |
| Supplementary content | 13 | Where possible, statements to the effect of “as described by ref [x]” were avoided. Protocols and/or materials cited in the text of the published study are included in supplementary materials or made available online via repositories. Cited recipes (e.g. commercial media formulations) are publicly available, without firewalls or requirements to purchase the product to verify the formulation. |  |
| Data availability | 14 | Where possible, raw data of environmental monitoring are archived in an on-line (freely accessible) repository, and/or provided in the supplementary information. |  |

* pH values have the dimension of 1 and are unit-less. However, when pH is defined, it is implicitly based on a concentration unit: for hydrogen ions. The type of pH standards used for calibration thus determines the pH scale.

** NBS and NIST pH scales are commonly used for physiology.
